# Effects of arousal and valence on center of pressure and ankle muscle activity during quiet standing

**DOI:** 10.1371/journal.pone.0297540

**Published:** 2024-04-18

**Authors:** Ryogo Takahashi, Naotsugu Kaneko, Hikaru Yokoyama, Atsushi Sasaki, Kimitaka Nakazawa

**Affiliations:** 1 Department of Life Sciences, Graduate School of Arts and Sciences, The University of Tokyo, Tokyo, Japan; 2 Institute of Engineering, Tokyo University of Agriculture and Technology, Tokyo, Japan; 3 The Miami Project to Cure Paralysis, University of Miami Miller School of Medicine, Miami, Florida, United States of America; University of L’Aquila Department of Clinical Sciences and Applied Biotechnology: Universita degli Studi dell’Aquila Dipartimento di Scienze Cliniche Applicate e Biotecnologiche, ITALY

## Abstract

Emotion affects postural control during quiet standing. Emotional states can be defined as two-dimensional models comprising valence (pleasant/unpleasant) and arousal (aroused/calm). Most previous studies have investigated the effects of valence on postural control without considering arousal. In addition, studies have focused on the center of pressure (COP) trajectory to examine emotional effects on the quiet standing control; however, the relationship between neuromuscular mechanisms and the emotionally affected quiet standing control is largely unknown. This study aimed to investigate the effects of arousal and valence on the COP trajectory and ankle muscle activity during quiet standing. Twenty-two participants were instructed to stand on a force platform and look at affective pictures for 72 seconds. The tasks were repeated six times, according to the picture conditions composed of arousal (High and Low) and valence (Pleasant, Neutral, and Unpleasant). During the task, the COP, electromyogram (EMG) of the tibialis anterior and soleus muscles, and electrocardiogram (ECG) were recorded. The heart rate calculated from the ECG was significantly affected by valence; the value was lower in Unpleasant than that in Neutral and Pleasant. The 95% confidence ellipse area and standard deviation of COP in the anterior-posterior direction were lower, and the mean power frequency of COP in the anterior-posterior direction was higher in Unpleasant than in Pleasant. Although the mean velocity of the COP in the medio-lateral direction was significantly lower in Unpleasant than in Pleasant, the effect was observed only when arousal was low. Although the EMG variables were not significantly affected by emotional conditions, some EMG variables were significantly correlated with the COP variables that were affected by emotional conditions. Therefore, ankle muscle activity may be partially associated with postural changes triggered by emotional intervention. In conclusion, both valence and arousal affect the COP variables, and ankle muscle activity may be partially associated with these COP changes.

## 1. Introduction

Emotion can elicit an appropriate behavioral response to handle environmental events that trigger it [1]. Emotion is fundamentally organized by two basic types of stimuli, appetitive or aversive, and each stimulus is associated with a different emotion and behavior. Appetitive stimuli are associated with pleasant emotions and underlain by approach behavior, whereas aversive stimuli are related to unpleasant emotions and withdrawal behavior [2–4].

Regarding the relationship between emotion and behavior, many researchers have investigated the effects of affective interventions on standing posture by emotionally eliciting pictures or videos [5–12]. Most emotional conditions were classified into the following categories: unpleasant, neutral, and pleasant. Substantial research has shown a smaller center of pressure (COP) sway (i.e., standard deviation, total path length, and sway area) or higher frequency power of COP in the unpleasant condition than in the neutral and pleasant conditions [5–11]. Moreover, heart rate reduction (also called “fear bradycardia”) concurrent with the COP changes described above in the unpleasant condition was reported [5, 6, 8, 9]. The simultaneous observation of reduced COP sway and fear bradycardia is physiologically interpreted as “freezing,” which serves to avoid detection by predators and to enhance perception when an intermediate-level predatory threat is detected [13]. However, the effect of pleasant stimuli on COP variables remains controversial. For example, some studies mentioned that pleasant emotions elicited no changes in COP variables (i.e., sway area, standard deviation, total path length, and mean power frequency) [5, 7–9], whereas others reported increased or reduced values of COP variables in the pleasant condition compared to those in the neutral condition [6, 11]. These studies focused on the influence of pleasantness, also called “valence,” on the standing posture. However, an emotional state can be defined as a two-dimensional model composed of valence (pleasant/unpleasant) and arousal (aroused/calm) [2, 14]. However, most studies did not set the same arousal scores across valence conditions [5–9, 11]. Although a part of them attempted to set uniform arousal scores between the unpleasant and pleasant conditions, the neutral condition was less aroused than the unpleasant and pleasant conditions [5]. Conversely, Horslen and Carpenter [12] investigated the effects of arousal, valence, and their interaction on quiet standing by setting arousal scores adequately; they identified a higher mean power frequency of COP in high-arousal than in low-arousal and no significant interaction effect of arousal and valence on COP. Mouras et al. [10] reported lower COP sway in sexual visual stimuli than that associated with other pleasant and neutral stimuli, indicating that COP variables are sensitive to arousal because sexual stimuli are the most aroused stimuli among pleasant stimuli. Moreover, the tendon reflex of the soleus muscle is affected by valence only when arousal is low [15]. Taken together, not only valence, but also arousal and their interaction may affect the motor control mechanism.

In the study by Horslen and Carpenter [12] on the effects of arousal and valence on, COP variables including standard deviation, mean power frequency, and mean position were used. These variables are often used to evaluate the postural control. Conversely, the effects of arousal, valence, and their interaction on the total path length (same as the mean velocity) are unknown, although they were affected by emotion in previous studies [7, 9]. Each COP variable represents different aspects of postural control, as mean velocity reflects the amount of postural control activity required to maintain standing balance, whereas standard deviation reflects postural stability [16]. Therefore, using different COP variables facilitates approaching postural control characteristics from multiple perspectives. Additionally, studies have focused on the relationship between emotion and standing posture in terms of the COP (i.e., sway area [5, 10, 11], standard deviation [5–8, 10, 12], total path length [7, 9], and mean power frequency [5, 6, 12]). Measuring physiological indices, such as electromyogram (EMG), in addition to COP variables, clarifies the mechanisms underlying behavioral changes.

Activities of the ankle plantar and dorsiflexor muscles play important roles in postural control during quiet standing [17]. In particular, the soleus muscle of the ankle dorsiflexor muscles constantly changes, depending on the COP trajectory [18]. Such postural control with ankle muscles is called the “ankle strategy,” and co-contraction of the ankle plantar and dorsiflexor muscles is observed for smaller COP sway in fear and anxiety of posture (i.e., postural threat) [19]. Hence, it is possible that the ankle muscle co-contraction is involved in the COP sway changes by emotional intervention. Focusing on ankle muscle activities in addition to COP would provide a better understanding of the influence of emotion on the standing posture.

In the current study, we aimed to investigate the emotional effects of arousal and valence on postural control during quiet standing from the perspective of the COP and ankle muscle activity. We hypothesized that COP variables are influenced by not only valence but also arousal or their interaction and that changes in COP variables are explained by ankle muscle activity.

## 2. Methods

### 2.1. Participants

Prior to the experiment, we conducted a power analysis to determine the required sample size using G*Power (ver. 3.1). Specifically, we referred a previous study that reported effects of arousal and valence on COP variables during quiet standing [12] and used the effect size of the COP variable (mean power frequency) affected by emotional states to calculate the sample size (effect size (ηp2): 0.151; α level: 0.05; power (1-β error probability): 0.95). As a result, the calculated necessary number of participants was twenty-two. According to the power analysis result, twenty-two healthy male volunteers were recruited. The mean ± standard deviation (SD) of age, weight, height, and BMI of the participants were 24.2 ± 3.6 years, 66.1 ± 10.4 kg, 173.0 ± 6.3 cm, and 22.0 ± 3.0, respectively. All participants provided written informed consent to participate in the study, and the experimental procedures were approved by the local ethics committee of the University of Tokyo (number:792). This study was performed in accordance with the Declaration of Helsinki (1964). Prior to the experiment, participants answered 21 questions on the Beck Depression Inventory-II [20] to assess their degree of depression; no participant had a depression score of > 17. This cut-off score was in line with that of a previous study [21].

### 2.2. Affective stimulus

The International Affective Picture System (IAPS) [22] was used for affective interventions. Self-Assessment Manikin (SAM) [23] values were attached to each picture as quantified values of arousal and valence. SAM values ranged from 1 to 9, where low values indicated low arousal or unpleasant valence and high values indicated high arousal or pleasant valence. Twelve pictures were selected for the following six conditions comprising two arousal conditions (High and Low) and three valence conditions (Unpleasant, Neutral, and Pleasant): (1) High-Unpleasant, (2) High-Neutral, (3) High-Pleasant, (4) Low-Unpleasant, (5) Low-Neutral, and (6) Low-Pleasant. We used the following pictures from the IAPS: (1) High-Unpleasant– 2703, 2811. 3030, 3110, 6022, 6313, 6360, 6560, 9252, 9630, 9902, 9921; (2) High-Neutral– 1114, 1120, 1310, 1321, 1617, 1726, 1931, 1932, 3302, 5940, 6900, 7640; (3) High-Pleasant– 1650, 2030, 4240, 4676, 5470, 7501, 8034, 8300, 8340, 8380, 8470, 9156; (4) Low-Unpleasant– 2053, 2095, 2141, 2205, 2750, 3301, 9007, 9181, 9220, 9265, 9340, 9571; (5) Low-Neutral– 1112, 1240, 1675, 2351, 2780, 2810, 4613, 5395, 6610, 7247, 7550, 9171; (6) Low-Pleasant– 1721, 1722, 1999, 2040, 2058, 2080, 2311, 2332, 5814, 5836, 7330, 7470.

### 2.3. Data collection

The ground reaction forces and moments were measured using a force plate at a sampling frequency of 1000 Hz. Although the first 11 participants stood on a force plate (TFG-4060-A, Tec Gihan, Japan), 11 others were recorded with another force plate embedded in a split-belt treadmill (Bertec, Columbus, OH, USA) because of mechanical problems. Before the electrodes were applied, the skin was cleaned with alcohol to reduce the impedance. EMG and electrocardiogram (ECG) data were collected by attaching two bipolar Ag/AgCl surface electrodes (Vitrode F-150S, Nihon Kohden, Tokyo, Japan) on the skin. EMG data were recorded bilaterally from the tibialis anterior (TA) and soleus muscles (SOL) in line with the SENIAM recommendation [24]. Two electrodes for the TA were placed at 1/3 of the line between the tip of the fibula and the tip of the medial malleolus, while those for the SOL were placed at 2/3 of the line between the medial condyles of the femur and the medial malleolus. The two electrodes were placed over the muscle belly with a 1 cm separation.

The ECG data were recorded using a lead II pattern with two electrodes. EMG and ECG signals were bandpass-filtered (5–1000 and 0.08–1000 Hz, respectively) and amplified (×1000) using a multichannel amplifier (MEG-6108, Nihon Kohden, Tokyo, Japan). All EMG and ECG data were recorded at a sampling frequency of 1000 Hz using an analog-to-digital (A/D) converter (Powerlab/16SP, AD Instruments, Castle Hill, Australia).

### 2.4. Procedures

The participants stood on a force plate with their feet shoulder-width apart, arms relaxed along the trunk, and looked at a 17-inch monitor. The participants’ toe and heel positions were marked on the force plate with tapes to ensure consistency of foot position across trials. The monitor was placed 1 m in front of the participants and its height was adjusted to the eye line. The experiment consisted of six blocks, and each block started with a 60 s preparatory phase followed by a 72 s intervention phase. A fixation cross was displayed in the preparatory phase and 12 affective pictures for each condition were displayed in each intervention phase. Each affective picture was displayed for 6 s successively, without any interval between the images. The experimental block order was randomized across participants. Two-minute breaks were set between the blocks. After the completion of the whole experiment, participants rated the subjective arousal and valence scores for each affective picture using SAM values. Manikins expressing emotional states corresponding to each score for arousal and valence were presented, and the participants chose the number that most appropriately reflected their feelings.

### 2.5. Data analysis

All signals were analyzed using a custom-written script in MATLAB (2021a, MathWorks Inc.). The preparatory phase was not analyzed, but the intervention phase was analyzed, as the phase was set to stabilize the standing posture. As two participants moved voluntarily at the end of the trials, these data were analyzed, except for the last 2 s.

The signals from the force plate were low-pass filtered at 5 Hz using a second-order Butterworth filter, and the COP in the anterior-posterior (AP) and medio-lateral (ML) directions were calculated. Using the COP data, the following four parameters were calculated for each condition: 95% confidence ellipse area (Ellipse area), SD, mean power frequency (MPF), and mean velocity (MV). The Ellipse area encloses approximately 95% of the COP trajectory and is considered an index of postural performance; the smaller the ellipse area, the better the performance [25]. The Ellipse area was calculated by fitting the principal component analysis method described in a previous study [26]. The SD, MV, and MPF were calculated separately for each axis. The SD reflects the variability of the COP amplitude [19] and provides information about the dynamic aspects of postural control [27]. The MPF indicates the average frequency within a power spectrum and reflects ankle stiffness [17, 28]. The MPF was calculated using Eq (1) [19]:

$$MPF\;\text{[Hz]}=\frac{\Sigma f∙P\left(f\right)}{\Sigma P\left(f\right)}$$ (1)

where *f* is the frequency of the COP signal and *P* is the power at the frequency. The MV is regarded as an indicator of the amount of control activity for postural control [16] and was calculated by dividing the total path length of the COP by the trial duration.

All EMG data were bandpass filtered at 15–500 Hz using a second-order Butterworth filter after subtraction of the DC bias to remove movement artifact [29, 30]. To quantify the muscle activity of the TA and SOL, the integrated EMG per second (TA iEMG and SOL iEMG) was calculated from the rectified EMG signal. Additionally, the iEMG ratio of the TA to SOL (TA/SOL ratio) was calculated as the activity ratio of the TA to SOL. To assess the degree of ankle muscle co-contraction, the co-contraction index (CI) described by Falconer and Winter [31] was calculated. Specifically, Eq (2) was used.

$$CI\;[\%]=\frac{2I_{ant}}{I_{total}}\times 100$$ (2)

where *I*_*ant*_ is the total antagonist muscle activity, calculated using Eq (3):

$$I_{ant}=\int_{t_{1}}^{t_{2}}{EMG}_{TA}\left(t\right)dt+\;\int_{t_{2}}^{t_{3}}{EMG}_{SOL}\left(t\right)dt$$ (3)

where *t*_1_ to *t*_2_ is the period in which the TA activity is smaller than the SOL activity and *t*_2_ to *t*_3_ is the period in which the SOL activity is smaller than the TA activity. *I*_*total*_ is the total muscle activity of the TA and SOL, which is calculated using Eq (4):

$$I_{total}=\int_{t_{1}}^{t_{3}}[{EMG}_{TA}+\;{EMG}_{SOL}]\;\left(t\right)dt$$ (4)

All EMG variables were averaged between the left and right legs. This is because our greatest interest was muscle activities related to anterior-posterior control. This averaging process was consistent with a previous study in which effects of postural threat on the quiet standing were examined [19]. All ECG data were low-pass filtered at 20 Hz using a second-order Butterworth filter. Next, R-waves were detected in the time-series data of the ECG to obtain R-R interval data. The mean heart rate of the entire intervention phase was then calculated using the R-R interval data.

### 2.6. Statistical analysis

The Shapiro-Wilk test was conducted to confirm the normal distribution of parameters. As the parameters showed non-normal distribution, all data were transformed using the aligned rank transformation (ART) procedure. Performing ART makes it possible to apply the analysis of variance (ANOVA) of parametric method [32]. Next, a two-way repeated-measures ANOVA was performed with arousal (High and Low) and valence (Unpleasant, Neutral, and Pleasant) as the within-subject factors. When a significant main effect or interaction was observed, we performed a post-hoc contrast test [33]. In addition, we calculated the Spearman correlation coefficients between the COP and EMG variables. All p-values were corrected using false discovery rate correction (Benjamini-Hochberg method [34]). The significance level for all tests was set at *p* < 0.05. All statistical comparisons were performed using R software package (ver. 4.1.2). The effect size for the main effect was reported as partial eta squared (*η*_*p*_^*2*^) when the ANOVA was conducted. For post-hoc tests, the effect size was reported as Cohen’s d (*d*).

## 3. Results

### 3.1. SAM ratings

**Fig 1:**
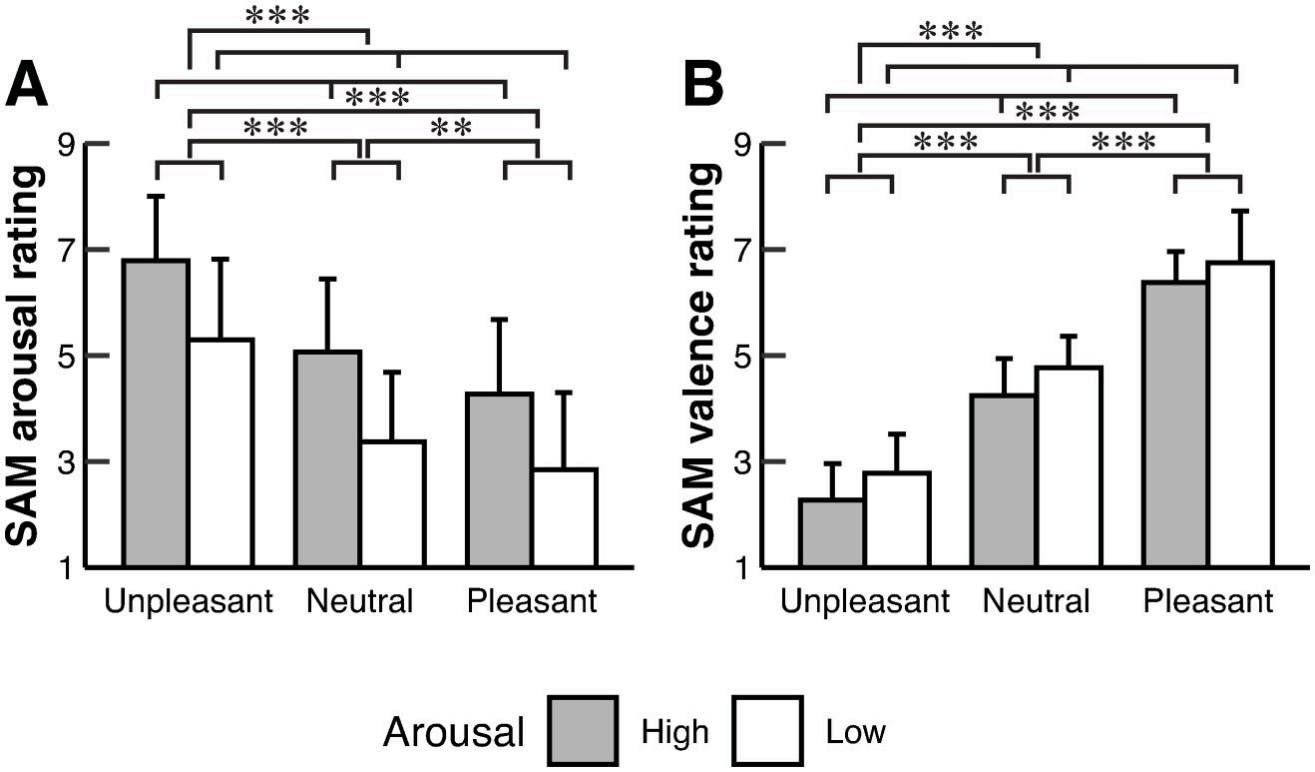
The mean values for (A) SAM arousal rating and (B) SAM valence rating in each condition. Error bars represent the SD. Asterisks indicate significant differences (** *p* < 0.01, *** *p* < 0.001), whereas *n*.*s*. indicates no significant differences. SAM arousal rating was significantly higher in High than Low. In addition, the SAM arousal rating score in Unpleasant was higher than those in Neutral and Pleasant, and that in Neutral was higher than that in Pleasant. Moreover, the SAM valence rating score in Unpleasant was lower than those in Neutral and Pleasant, and that in Neutral was lower than that in Pleasant.

Two-way ART-ANOVA (two arousals × three valences) revealed a significant main effect of arousal on SAM arousal rating, and the rating score was higher in High than Low (*p* < 0.001) [Tables 1 and 2; Fig 1A]. Furthermore, the two-way ART-ANOVA revealed a significant main effect of valence on SAM arousal ratings (*p* < 0.001) [Table 2]. Post-hoc contrast tests revealed that the rating score was higher in Unpleasant than Neutral (*p* < 0.001) and Pleasant (*p* < 0.001) [Tables 1 and 3; Fig 1A]. Moreover, the rating score was higher in Neutral than Pleasant (*p* = 0.008). Meanwhile, the two-way ART-ANOVA revealed a significant main effect of arousal on SAM valence rating, and the rating score was lower in High than Low (*p* < 0.001) [Tables 1 and 2; Fig 1B]. In addition, the two-way ART-ANOVA revealed a significant main effect of valence on SAM valence rating (*p* < 0.001) [Table 2]. Post-hoc tests revealed that the rating score in Unpleasant was lower than those in Neutral (*p* < 0.001) and Pleasant (*p* < 0.001), and that in Neutral was lower than that in Pleasant (*p* < 0.001) [Tables 1 and 3; Fig 1B].

**Table 1:** Summary of the mean values (mean ± SD) of the measured variables in each condition.

	Conditions (Arousal-Valence)					
	High-Unpleasant	Low-Unpleasant	High-Neutral	Low-Neutral	High-Pleasant	Low-Pleasant
*SAM ratings*						
SAM arousal	6.8 ± 1.2	5.3 ± 1.5	5.1 ± 1.4	3.4 ± 1.3	4.3 ± 1.4	2.8 ± 1.5
SAM valence	2.3 ± 0.7	2.8 ± 0.7	4.2 ± 0.7	4.8 ± 0.6	6.4 ± 0.6	6.8 ± 1.0
*COP variables*						
Ellipse area [mm^2^]	76.7 ± 33.3	74.9 ± 45.2	93.8 ± 74.3	99.7 ± 107.8	93.8 ± 57.9	124.3 ± 95.6
SD-AP [mm]	3.16 ± 1.07	2.88 ± 0.89	3.27 ± 1.19	3.40 ± 1.21	3.54 ± 1.21	4.06 ± 2.07
SD-ML [mm]	1.36 ± 0.43	1.37 ± 0.59	1.42 ± 0.68	1.45 ± 0.74	1.43 ± 0.55	1.60 ± 0.63
MPF-AP [Hz]	0.19 ± 0.07	0.23 ± 0.08	0.19 ± 0.07	0.19 ± 0.07	0.17 ± 0.06	0.17 ± 0.07
MPF-ML [Hz]	0.25 ± 0.10	0.26 ± 0.16	0.29 ± 0.12	0.26 ± 0.15	0.29 ± 0.19	0.27 ± 0.20
MV-AP [mm/s]	4.27 ± 1.10	4.30 ± 1.05	4.47 ± 1.29	4.26 ± 1.22	4.35 ± 1.32	4.42 ± 1.11
MV-ML [mm/s]	2.33 ± 0.54	2.24 ± 0.52	2.42 ± 0.59	2.39 ± 0.66	2.28 ± 0.48	2.52 ± 0.69
*EMG variables*						
TA iEMG [mV]	1.83 ± 0.80	1.87 ± 0.91	1.67 ± 0.51	1.78 ± 0.86	1.84 ± 0.73	1.81 ± 0.77
SOL iEMG [mV]	19.00 ± 9.05	18.75 ± 9.87	18.89 ± 7.90	19.13 ± 10.25	18.45 ± 8.89	18.30 ± 7.62
TA/SOL ratio	0.11 ± 0.05	0.12 ± 0.09	0.10 ± 0.05	0.12 ± 0.11	0.12 ± 0.07	0.12 ± 0.08
CI [%]	18.0 ± 6.6	19.1 ± 8.4	16.7 ± 5.8	17.7 ± 6.7	18.3 ± 7.0	17.5 ± 6.4
*Heart rate*						
Heart rate [bpm]	78.8 ± 11.5	79.1 ± 11.2	80.1 ± 11.3	80.3 ± 12.2	80.0 ± 11.1	80.6 ± 12.0

**Table 2:** Summary of two-way ART-ANOVA (arousal × valence) of the measured variables. Significant *p-values* (< 0.05) are boldfaced.

	Arousal main effect			Valence main effect			Arousal × Valence interaction		
	*F*_(1, 105)_	*p-value*	*η*_*p*_^*2*^	*F*_(2, 105)_	*p-value*	*η*_*p*_^*2*^	*F*_(2, 105)_	*p-value*	*η*_*p*_^*2*^
*SAM ratings*									
SAM arousal	69.2	**< 0.001**	0.397	60.2	**< 0.001**	0.534	0.303	0.740	0.006
SAM valence	17.8	**< 0.001**	0.145	421	**< 0.001**	0.889	0.332	0.718	0.006
*COP variables*									
Ellipse area	0.939	0.335	0.009	3.98	**0.021**	0.070	1.49	0.230	0.028
SD-AP	0.402	0.527	0.107	5.81	**0.004**	0.107	1.25	0.292	0.023
SD-ML	0.485	0.488	0.004	1.35	0.264	0.025	0.760	0.470	0.014
MPF-AP	1.35	0.248	0.013	3.68	**0.029**	0.065	1.13	0.326	0.021
MPF-ML	1.84	0.178	0.017	0.600	0.551	0.011	0.094	0.911	0.001
MV-AP	0.005	0.942	0.000	0.175	0.840	0.003	1.29	0.279	0.024
MV-ML	0.335	0.564	0.003	1.46	0.237	0.027	3.20	**0.045**	0.057
*EMG variables*									
TA iEMG	0.062	0.804	0.001	1.24	0.295	0.023	0.292	0.747	0.006
SOL iEMG	0.799	0.374	0.008	0.583	0.560	0.011	0.031	0.970	0.001
TA/SOL ratio	1.09	0.299	0.010	1.25	0.291	0.023	0.744	0.478	0.014
CI	0.004	0.953	0.000	1.43	0.244	0.027	1.17	0.316	0.022
*Heart rate*									
Heart rate	0.587	0.445	0.006	7.92	**< 0.001**	0.131	0.286	0.752	0.005

**Table 3:** Summary of the post-hoc contrast tests following the main effects of valence. Significant *p-values* (< 0.05) are boldfaced.

Post-hoc contrast tests following valence main effect									
	Unpleasant vs Neutral			Neutral vs Pleasant			Pleasant vs Unpleasant		
	*t*_*(105)*_	*p-value*	*d*	*t*_*(105)*_	*p-value*	*d*	*t*_*(105)*_	*p-value*	*d*
SAM arousal	7.85	**< 0.001**	1.27	2.72	**0.008**	0.440	10.6	**< 0.001**	1.71
SAM valence	14.4	**< 0.001**	2.79	14.6	**< 0.001**	2.83	29.0	**< 0.001**	5.62
Ellipse area	0.841	0.402	0.118	1.91	0.088	0.267	2.75	**0.021**	0.385
SD-AP	1.63	0.107	0.252	1.78	0.107	0.275	3.41	**0.003**	0.527
MPF-AP	1.60	0.168	0.293	1.09	0.276	0.200	2.70	**0.025**	0.493
Heart rate	3.16	**0.002**	0.148	0.511	0.611	0.024	3.67	**< 0.001**	0.172

### 3.2. COP variables

**Fig 2:**
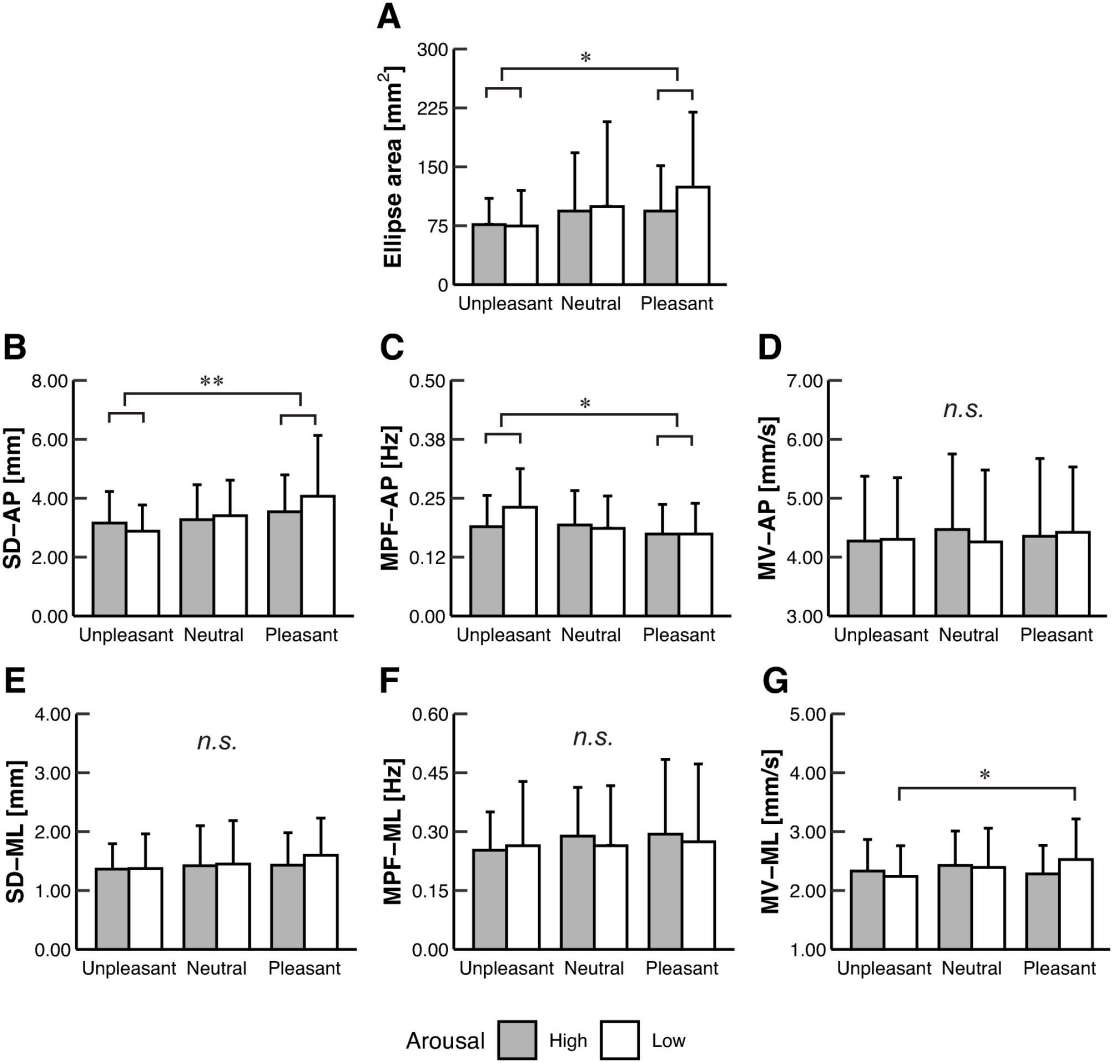
The mean values for (A) Ellipse area, (B) SD-AP, (C) MPF-AP, and (D) MV-AP, (E) SD-ML, (F) MPF-ML, and (G) MV-ML in each condition. Error bars represent the SD. Asterisks indicate significant differences (* *p* < 0.05, ** *p* < 0.01), whereas *n*.*s*. indicates no significant differences. The Ellipse area and SD-AP were significantly lower in Unpleasant than Pleasant. The MPF-AP was significantly higher in Unpleasant than Pleasant. The MV-ML was significantly lower in Unpleasant than Pleasant only when arousal was Low. No significant differences were observed in the MV-AP, SD-ML, or MPF-ML across the conditions.

The two-way ART-ANOVA showed significant main effects of valence on the Ellipse area (*p* = 0.021), SD-AP (*p* = 0.004), and MPF-AP (*p* = 0.029) [Table 2]. Post-hoc tests revealed that the Ellipse area was significantly smaller in Unpleasant than Pleasant (*p* = 0.021) [Tables 1 and 3; Fig 2A], and SD-AP was significantly lower in Unpleasant than Pleasant (*p* = 0.003) [Fig 2B]. MPF-AP was significantly higher in Unpleasant than Pleasant (*p* < 0.025) [Tables 1 and 3; Fig 2C]. The two-way ART-ANOVA revealed a significant interaction on the MV-ML (*p* = 0.045) [Table 2]. A simple main effect of valence was observed when arousal was Low (*p* = 0.035) [Table 4], and the post-hoc contrast tests revealed that the MV-ML was lower in Unpleasant than Pleasant (*p* < 0.021) [Tables 1 and 4; Fig 2G].

**Table 4:** Summary of the simple main effects of arousal and valence, followed by post-hoc contrast tests. Significant *p-values* (< 0.05) are boldfaced.

Arousal simple main effects in each valence condition											
	Unpleasant				Neutral				Pleasant		
	*t*_*(21)*_	*p-value*	*d*		*t*_*(21)*_	*p-value*	*d*		*t*_*(21)*_	*p-value*	*d*
MV-ML	1.55	0.226	0.226		0.936	0.936	0.014		2.15	0.108	0.341
Valence simple main effects in each arousal condition											
			High				Low				
			*F*_(2, 42)_	*p-value*	*η*_*p*_^*2*^		*F*_(2, 42)_	*p-value*	*η*_*p*_^*2*^		
MV-ML			0.374	0.863	0.017		5.592	**0.035**	0.210		
Post-hoc contrast tests following valence simple main effect in Low											
	Unpleasant vs Neutral				Neutral vs Pleasant				Pleasant vs Unpleasant		
	*t*_*(42)*_	*p-value*	*d*		*t*_*(42)*_	*p-value*	*d*		*t*_*(42)*_	*p-value*	*d*
MV-ML	0.841	0.402	0.118		1.91	0.088	0.267		2.75	**0.021**	0.385

### 3.3. EMG variables

**Fig 3:**
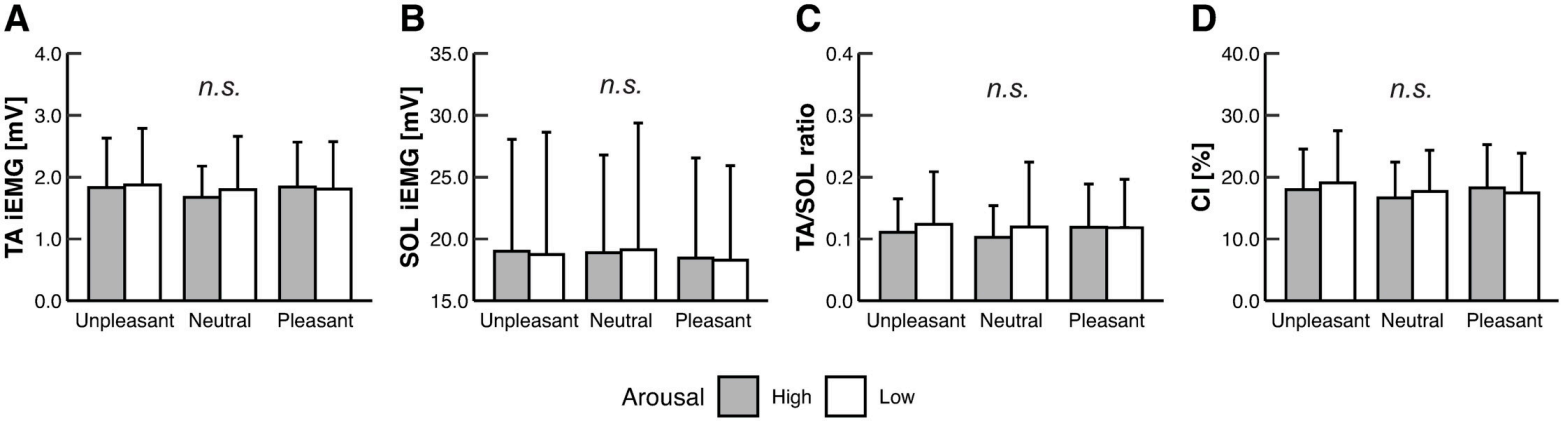
The mean values for (A) TA iEMG, (B) SOL iEMG, (C) TA/SOL ratio, and (D) CI in each condition. Error bars represent SD. *n*.*s*. indicates no significant difference. No significant main effects or interactions were observed for any of the EMG variables.

In contrast to the COP results, the two-way ART-ANOVA did not show any main effect or interaction on all EMG variables [Tables 1 and 2; Fig 3].

### 3.4. Correlation coefficient between the COP and EMG variables

Spearman correlation coefficients revealed that the TA iEMG was significantly correlated with the Ellipse area, SD-AP, and MV-ML [Table 5]. The SOL iEMG was significantly correlated with the MPF-ML, MV-AP, and MV-ML. The TA/SOL ratio was significantly correlated with the Ellipse area, SD-AP, SD-ML, MPF-AP, and MV-ML. The CI was significantly correlated with the Ellipse area, SD-AP, SD-ML, MV-AP, and MV-ML.

**Table 5:** Summary of correlation coefficients between the COP and EMG variables. Correlation coefficients with significant *p-values* are in boldface (* *p <* 0.05, ** *p <* 0.01, *** *p <* 0.001).

	Ellipse area	SD-AP	SD-ML	MPF-AP	MPF-ML	MV-AP	MV-ML
TA iEMG	0.254**	0.262**	0.173	−0.102	0.132	0.136	0.488***
SOL iEMG	−0.151	−0.159	−0.131	0.104	0.212*	−0.202*	0.210*
TA/SOL ratio	0.334***	0.350***	0.280**	−0.200*	−0.154	0.281**	0.066
CI	0.312**	0.320**	0.267**	−0.187	−0.168	0.255**	0.038

### 3.5. Heart rate

**Fig 4:**
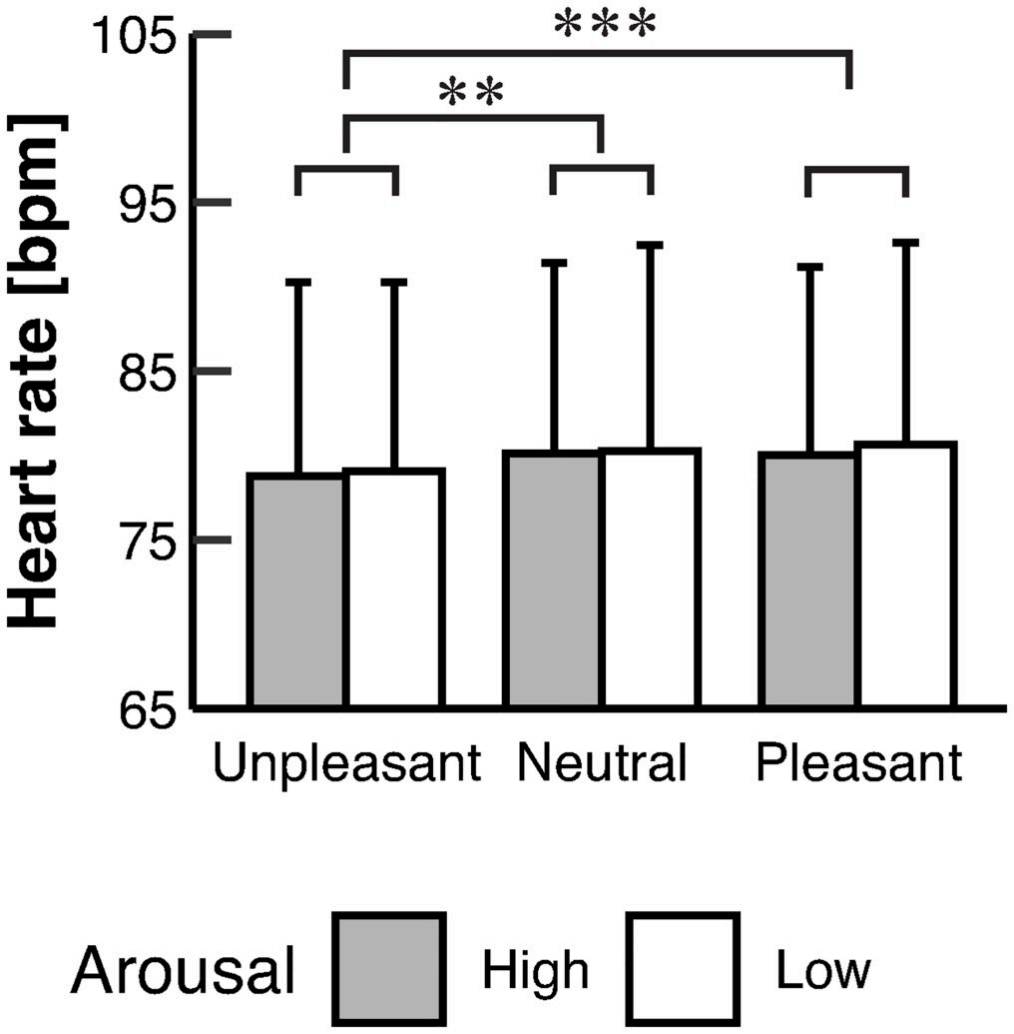
The mean value for the heart rate in each condition. Error bars represent SD. Asterisks indicate significant differences (** *p* < 0.01; *** *p* < 0.001). Heart rate in Unpleasant was significantly lower in those in Neutral and Pleasant.

The two-way ART-ANOVA revealed a significant main effect of valence on heart rate (*p* < 0.001) [Table 2]. The post-hoc contrast tests revealed lower heart rate in Unpleasant than those in Neutral (*p* = 0.002) and Pleasant (*p* < 0.001) [Tables 1 and 3; Fig 4].

## 4. Discussion

The present study found that valence affected the Ellipse area, SD-AP, and MPF-AP of the COP. The Ellipse area and SD-AP were lower in Unpleasant than Pleasant. The MPF-AP was higher in Unpleasant than Pleasant. These results conform with those of previous studies that identified COP sway reduction in the unpleasant emotions compared with neutral or pleasant emotions, irrespective of arousal scores [5–9, 11]. Importantly, the current study demonstrated that the MV-ML was lower in Unpleasant than Pleasant, indicating that the MV-ML was influenced by valence only when arousal was low. With respect to ankle muscle activity, our results revealed no significant differences in the EMG variables among any of the conditions. However, some EMG variables were significantly correlated with the COP variables that were affected by emotional intervention. The observed results regarding the COP and EMG variables are discussed below.

### 4.1. Effects of arousal and valence on COP variables

In the present study, smaller sway and higher frequency power of the COP in the AP direction were observed in the unpleasant emotions compared with the pleasant emotions [Fig 2A–2C]. Moreover, we observed a reduction in heart rate in the unpleasant emotions compared with those in the neutral and pleasant emotions [Fig 4]. The heart rate reduction due to the presentation of the unpleasant stimuli is called fear bradycardia. Concurrent observations of COP sway reduction and fear bradycardia are considered freezing [13]. Freezing is a preparatory state for eliciting subsequent definitive fight or flight behavior via both sympathetic and parasympathetic nervous activities, and the enhancement of threat perception and motor inhibition is observed [13]. Therefore, immobility seemingly emerged as defensive behavior when participants saw the unpleasant pictures in the present study. When humans face a threat, the ventrolateral periaqueductal gray, which receives input from the amygdala, modulates many physiological responses accompanying freezing [13]. Moreover, COP changes in the unpleasant condition might be related to the activity of the basal ganglia in addition to the freezing pathway. The basal ganglia have cortical and subcortical loops and are involved in motor, emotional, and cognitive processes [35]. The striatum receives dopamine release from the substantia nigra pars compacta and the ventral tegmental area and adjusts output from the basal ganglia [36]. The mesolimbic dopamine system is affected by valence [37]. Therefore, activity modulation of the basal ganglia, which is attributed to the mesolimbic dopamine system, may support valence-dependent COP changes.

Alternatively, the MV-ML of the COP was reduced in Unpleasant only when arousal was low [Fig 2G]. Physiological indices have revealed variable effects of valence on the motor control system depending on the arousal score. Bonnet et al. reported that the tendon reflex of the SOL muscle is modulated by valence when arousal is low [15]. Focusing on the brain, regions that are related to arousal (e.g., amygdala) and those related to valence (e.g., orbitofrontal cortex, mesolimbic dopamine system) are not independent but interact with each other [37]. Hence, the interaction among brain regions may be related to changes in the COP velocity according to arousal and valence. In contrast to the SD and MPF of the COP that indicated changes in the AP direction, the MV was changed in the ML direction. In general, AP sway is primarily controlled by the ankle muscles, whereas ML sway is mainly controlled by the hip muscles [38]. As such, AP and ML sway may be controlled by different neuromuscular control strategies [38]. Therefore, the interaction between arousal and valence may modulate the neuromuscular control strategies associated with ML control. Regarding the current results pertaining to the COP change in the ML direction, the detailed mechanisms underlying this change are important issues to be addressed in the future. Moreover, the neural mechanism that affects COP variables in both the AP and ML directions remains unclear and further investigation is required.

### 4.2. Ankle muscle activity related to COP changes

The current study revealed no significant differences in any EMG variables between the conditions [Fig 3]. However, some EMG variables were significantly correlated with the COP variables that were affected by emotional intervention (i.e., Ellipse area, SD-AP, MPF-AP, and MV-ML) [Table 5]. In particular, the MV-ML was moderately correlated with the TA iEMG and was weakly correlated with SOL iEMG as well. Namely, lower MV-ML of the COP in the unpleasant emotions than the pleasant emotions may be partially associated with lower TA and SOL activities. The TA and SOL have anatomical roles in ankle inversion as well as dorsiflexion or plantarflexion, and ankle inversion is associated with COP oscillation in the ML direction [39]. Considering that the MV of the COP reflects the amount of control activity required to maintain postural balance [16], it seems reasonable that the MV-ML and the TA and SOL iEMG have significant correlations. On the other hand, the Ellipse area and SD-AP were significantly correlated with the TA/SOL ratio, CI, and TA iEMG, which may indicate that smaller COP sway in the AP direction in the unpleasant emotions than the pleasant emotions would be associated with lower ankle co-contraction derived from lower TA activity. Previous studies have shown inconsistent results on how ankle co-contraction affects COP sway [40, 41]. During postural threat as participants stand on a height surface, ankle co-contraction leads to smaller postural sway to stabilize the posture [40]. In contrast, an ankle muscle co-contraction task without any goal of postural steadiness resulted in greater postural sway [41]. The present study probably did not require any consciousness of trying to stabilize posture because participants merely looked at affective pictures, and ankle co-contraction might have increased COP sway. The MPF-AP was weakly correlated with the TA/SOL ratio but not with each muscle activity (i.e., The TA and SOL iEMG); therefore, the relationship between the MPF-AP and the TA and SOL activities seems weak.

It is possible that no significant difference in the EMG variables among the emotional conditions and moderate or weak correlations between the COP and EMG variables could be attributed to muscle activities other than the TA and SOL. Although we measured the TA and SOL as ankle muscles, other ankle muscles (e.g., the medial gastrocnemius and peroneus longus muscles) play a role in regulating COP oscillation. In addition, quiet standing is a whole-body movement as fluctuations of the hip and knee are not negligible [42], and muscle activities other than the ankle muscles would be related to postural changes [43]. Therefore, in the present study, factors other than ankle muscle activity may be related to changes in the COP. Specifically, considering the possibility of freezing in the unpleasant emotions, proximal muscle (e.g., back and neck muscles) tone might have increased [44]. Additionally, Ciria et al. [45] identified a smaller head sway in the unpleasant emotions than that in the pleasant emotions during quiet standing. Thus, not only the lower body but also the upper body could be involved in COP changes. In addition, the influence of body movements associated with the modulation of the autonomic nervous system must be considered. Hagio et al. [46] reported that reduced COP sway during standing with a cognitive load was attributed to smaller thoracic movement by respiration [46]. The current study confirmed autonomic nervous system changes by observing changes in heart rate; accordingly, the possibility that body movements associated with heartbeats and respiration had an impact on COP should be considered.

### 4.3. Limitations

The present study had several limitations. First, we did not measure the physiological indices that reflect arousal. In this study, we confirmed the main effect of valence but not arousal on the heart rate. The results were consistent with a psychophysiological study [2], which suggests that valence was adequately manipulated. In contrast, the present study used SAM measurements and showed that SAM arousal ratings were significantly affected by valence in addition to arousal [Table 2; Fig 1A]. However, we did not measure a physiological index that changes according to arousal state (e.g., electrodermal activity [2]). In the future, the objective (physiological) index should be measured to confirm the adequate manipulation of arousal. Second, the current study did not measure the anxiety characteristics of the participants, although the participants with the depression were excluded. Previous studies have shown an association between postural balance and anxiety levels [47, 48]. For example, individuals with posttraumatic stress disorder who have high anxiety have increased postural sway while viewing unpleasant pictures, while healthy individuals have decreased postural sway [48]. Thus, anxiety could have affected postural control in this study.

## 5. Conclusions

We investigated the emotional effects of arousal and valence on postural control during quiet standing from the perspective of the COP and ankle muscle activities. Our results revealed that postural control was affected by arousal and valence during quiet standing. Specifically, smaller sway and higher frequency power of the COP in the AP direction were observed in unpleasant emotions compared with pleasant emotions. More importantly, the COP velocity in the ML direction was lower in the unpleasant emotions than the pleasant emotions. However, this effect was observed only when the arousal was low. These results suggest that the COP variables are influenced by both arousal and valence. Although the EMG variables were not significantly affected by emotional conditions, some EMG variables were significantly correlated with the COP variables that were affected by emotional conditions. Therefore, ankle muscle activity may be partially associated with postural changes triggered by emotional intervention.
